# Diagnostic Snapshot: Dyspnea in the Oncology Patient

**Published:** 2012-01-01

**Authors:** Robin Sommers

**Affiliations:** From Dana-Farber Cancer Institute, Boston, Massachusetts

**History**

Ms. G.M. is a 79-year-old woman who was diagnosed with esophageal cancer in June 2008, at which time she underwent neoadjuvant chemoradiation. This was followed by a three-hole esophagectomy with a thorascopic right chest dissection in May 2009, with complete pathologic response. Unfortunately, in May 2010 she was found to have a recurrence on chest CT; a CT-guided biopsy demonstrated metastatic carcinoma. Ms. G.M. underwent a video-assisted thoracic surgery wedge resection, and pathology revealed a squamous cell carcinoma consistent with her prior esophageal cancer. Subsequently, she developed another recurrence in the right upper lobe of the lung with mediastinal lymphadenopathy consistent with recurrent esophageal cancer. She began systemic chemotherapy with the FOLFOX (fluorouracil, leucovorin, oxalipatin) regimen.

## Chief Complaint

Following three cycles of FOLFOX, Ms. G.M. presented to the clinic with a chief complaint of shortness of breath as well as facial and arm swelling. The IV team nurses were unable to elicit a blood return from her portacath.

## Review of Systems

Ms. G.M. reported weakness and increasing shortness of breath over 2 weeks, as well as intermittent nausea, vomiting, and constipation following her last cycle of chemotherapy. On physical examination, vital signs were notable for temperature 97.5°F, pulse 89, blood pressure 140/80 mm Hg, respiratory rate 18, and O_2_ saturation of 97% on room air. Chest exam was notable for bibasilar crackles with evidence of dilated chest veins. Ms. G.M. underwent a vascular flow study (see Figure 1), and a chest CT scan with contrast had been obtained following vascular access placement (see Figure 2).

## Choose the correct diagnosis:

- A: PULMONARY EMBOLI
- B: PLEURAL EFFUSION
- C: SUPERIOR VENA CAVA SYNDROME

**Scroll down for correct answer.** 

**Figure:**
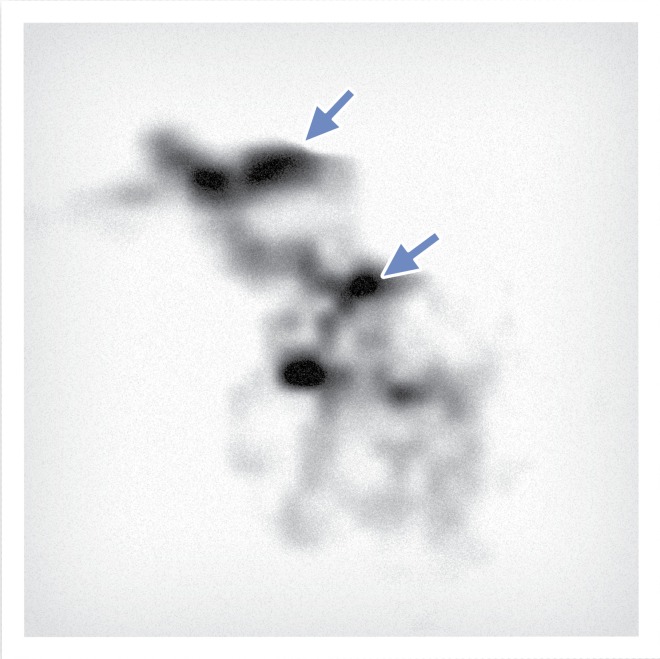


**Figure:**
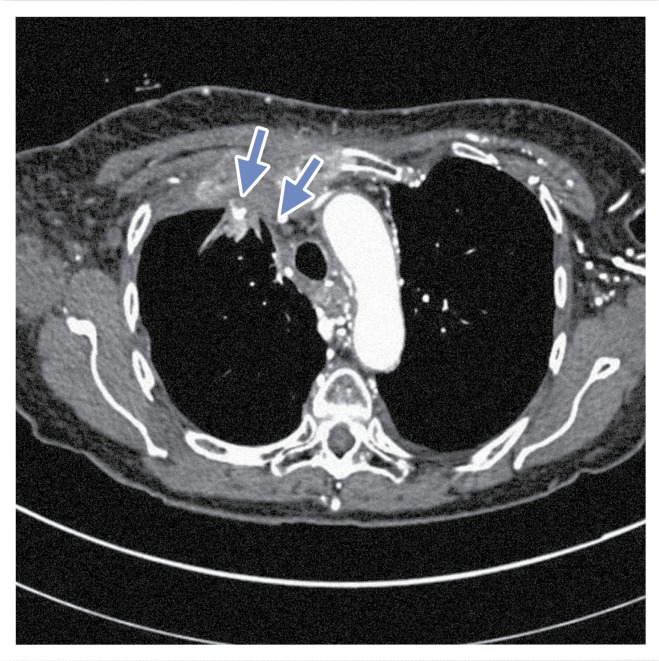


## Correct Answer 

***Superior vena cava syndrome (SVCS)***  results from the obstruction of the superior vena cava (SVC), commonly due to malignant tumors; other etiologies may include fibrosing mediastinitis and thrombosis from indwelling central venous access devices (CVAD; Lewis, Hendrickson, & Moynihan, 2011). Regardless of etiology, dyspnea is the most common symptom of SVCS (Bell, Woods, & Levi, 1986). When the SVC is obstructed, collateral vessels develop to accommodate diverted blood flow from the SVC, often taking several weeks (Wilson, Detterbeck, & Yahalom, 2007). The severity of symptoms depends on the acuity and speed of onset of the obstruction. Often there is a constellation of symptoms, most commonly including edema of the face, neck, and arm; dyspnea; cough; and dilated chest veins. Dysphagia, stridor, hoarseness, confusion, and syncope may also be present (Wan & Bezjak, 2009). Catheter thrombosis should be suspected when there is evidence of catheter dysfunction with accompanying signs and symptoms of facial, neck, or arm swelling (Nakazaw, 2010).

## Explanation of Incorrect Answers

**Pleural effusion**  is an abnormal collection of fluid in the pleural space (Mulroy, 2008). There are a number of causes for pleural effusions, all related to a disease process. Presenting signs and symptoms may include cough, shortness of breath, pleuritic chest pain, decreased or absent breath sounds, friction rub, or dullness on percussion. The pleural effusion may be categorized as either transudative or exudative. Transudative pleural effusions are essentially caused by protein-free fluid leaking from the capillaries into the pleural space, while exudative pleural effusions are concentrated with proteins, and may be caused by drug toxicity, inflammation, or blockage of blood vessels (Mertin, Sawatzky, & Diehl-Jones, 2009).

**Acute pulmonary embolism**  is characterized by a sudden unexplained dyspnea on exertion or at rest that is generally acute in onset; this is the most frequently reported symptom in patients with pulmonary embolism with no prior cardiopulmonary disease (Stein et al., 2007). Other signs include tachypnea, tachycardia, jugular venous distention, decreased breath sounds, cough, and pleuritic chest pain. Risk factors may include immobilization, surgery within 3 months, coronary artery disease, myocardial infarction, heart failure, malignancy, stroke, paresis or paralysis, central venous instrumentation, thrombophlebitis, and a prior history of pulmonary embolism (Stein & Matta, 2010).

## Management

Superior vena cava syndrome is often diagnosed clinically; however, diagnostic studies are often warranted when the exact cause of the obstruction is unknown. While the gold standard for localizing SVC obstruction is venography, chest CT and MRI may be useful in diagnosing the cause and guiding treatment planning (Lewis et al., 2011).

Management of SVCS depends on the cause of the compression. In Ms. G.M.’s case, the vascular flow study demonstrated central venous thrombosis involving the brachiocephalic veins with evidence of extensive collaterals and SVC and/or obstruction of the SVC, which could also be the result of compression of the chest wall mass. CT scan of the chest demonstrated both thrombosis and compression from the mass.

The goals of management for SVCS associated with malignancy are to alleviate symptoms and treat the underlying cause. Management of SVCS may include angioplasty, stenting, and thrombolysis (Baskin et al., 2009). For management of suspected CVAD portacath thrombosis, alteplase (Cathflow, Activase) may be used to restore blood flow. Alteplase is an FDA-approved agent for clearing CVAD occlusions (Genentech, 2005). Ms. G.M. had clinical evidence of improvement in symptoms following the initiation of therapeutic dalteparin (Fragmin), and as such, vascular medicine deferred endovascular stenting.

## Follow-Up

Ms. G.M. was readmitted for recurrent symptoms of SVCS 6 days after discharge. Vascular angiography demonstrated a segment of right subclavian vein occlusion and new thrombosis of the left subclavian vein. She underwent successful endovascular stent placement within the left subclavian vein with improvement in symptoms. The SVC was not stented as her CVAD would have had to have been removed. Endovascular treatment is a viable option for patients with SVCS given its high success rate in symptom relief, decreased time to SVC obstruction relapse, and improvement in overall survival (Zarogoulidis et al., 2011).
